# Effect of Al_2_TiO_5_ Content and Sintering Temperature on the Microstructure and Residual Stress of Al_2_O_3_–Al_2_TiO_5_ Ceramic Composites

**DOI:** 10.3390/ma14247624

**Published:** 2021-12-11

**Authors:** Kunyang Fan, Wenhuang Jiang, Jesús Ruiz-Hervias, Carmen Baudín, Wei Feng, Haibin Zhou, Salvador Bueno, Pingping Yao

**Affiliations:** 1 School of Mechanical Engineering, Chengdu University, Chengdu 610106, China; J15983027227@163.com (W.J.); fengwei@cdu.edu.cn (W.F.); 2 Sichuan Province Engineering Technology Research Center of Powder Metallurgy, Chengdu University, Chengdu 610106, China; 3 Materials Science Department, Universidad Politécnica de Madrid, E.T.S.I. Caminos, Canales y Puertos, C/Profesor Aranguren s/n, 28040 Madrid, Spain; jesus.ruiz@upm.es; 4 Instituto de Cerámica y Vidrio, Consejo Superior de Investigaciones Científicas (CSIC), Kelsen 5, 28049 Madrid, Spain; cbaudin@icv.csic.es; 5 State Key Laboratory of Powder Metallurgy, Central South University, Changsha 410083, China; zhbtc22@126.com (H.Z.); yaopingpingxx@sohu.com (P.Y.); 6 Department of Chemical, Environmental and Materials Engineering, Campus Las Lagunillas, Universidad de Jaén, s/n, 23071 Jaen, Spain; jsbueno@ujaen.es

**Keywords:** Al_2_TiO_5_, ceramics, crystal structure, residual stresses, neutron diffraction

## Abstract

A series of Al_2_O_3_–Al_2_TiO_5_ ceramic composites with different Al_2_TiO_5_ contents (10 and 40 vol.%) fabricated at different sintering temperatures (1450 and 1550 °C) was studied in the present work. The microstructure, crystallite structure, and through-thickness residual stress of these composites were investigated by scanning electron microscopy, X-ray diffraction, time-of-flight neutron diffraction, and Rietveld analysis. Lattice parameter variations and individual peak shifts were analyzed to calculate the mean phase stresses in the Al_2_O_3_ matrix and Al_2_TiO_5_ particulates as well as the peak-specific residual stresses for different *hkl* reflections of each phase. The results showed that the microstructure of the composites was affected by the Al_2_TiO_5_ content and sintering temperature. Moreover, as the Al_2_TiO_5_ grain size increased, microcracking occurred, resulting in decreased flexure strength. The sintering temperatures at 1450 and 1550 °C ensured the complete formation of Al_2_TiO_5_ during the reaction sintering and the subsequent cooling of Al_2_O_3_–Al_2_TiO_5_ composites. Some decomposition of AT occurred at the sintering temperature of 1550 °C. The mean phase residual stresses in Al_2_TiO_5_ particulates are tensile, and those in the Al_2_O_3_ matrix are compressive, with virtually flat through-thickness residual stress profiles in bulk samples. Owing to the thermal expansion anisotropy in the individual phase, the sign and magnitude of peak-specific residual stress values highly depend on individual *hkl* reflection. Both mean phase and peak-specific residual stresses were found to be dependent on the Al_2_TiO_5_ content and sintering temperature of Al_2_O_3_–Al_2_TiO_5_ composites, since the different developed microstructures can produce stress-relief microcracks. The present work is beneficial for developing Al_2_O_3_–Al_2_TiO_5_ composites with controlled microstructure and residual stress, which are crucial for achieving the desired thermal and mechanical properties.

## 1. Introduction

Aluminum titanate (AT (Al_2_TiO_5_)) is a compound with low thermal expansion, excellent thermal shock resistance, and low thermal conductivity. In view of these properties, it is a suitable second-phase candidate for improving the thermal and mechanical properties of alumina (Al_2_O_3_)-based ceramic systems [1,2]. Alumina–AT (A–AT) ceramics have attracted considerable attention as flaw-tolerant ceramics in thermal and structural applications, such as thermal insulation liners, diesel particulate filters, vehicle emissions control, and high-temperature flue gas filtration supports, because of their improved flaw tolerance, toughness, and superior thermal properties [3,4,5,6]. In addition to the typical investigation of mechanical properties, such as flaw tolerance, crack resistance, and thermal shock resistance, many studies have focused on the reinforcement mechanisms of A–AT ceramics [7,8,9]. The key factors presumed to be responsible for the toughness and improved flaw tolerance of ceramic composites are residual stresses [7].

High tensile and/or compressive residual stresses are expected to develop in alumina-based composites owing to the thermal expansion mismatch among different phases and thermal expansion anisotropy in each phase during cooling after sintering [10]. In the micro-mechanical interaction between matrix and reinforcing particles, residual stresses appear to perform an important function in the mechanical properties of A–AT composites. Padture et al. [11] and Asmi et al. [12] showed that the addition of AT to alumina could improve flaw tolerance and crack-growth resistance. They found that this resulted from the existence of residual stresses arising from the thermal mismatch between alumina and AT. Skala et al. [7] reported that the residual stresses in A–AT composites are responsible for a wide variety of possible toughening mechanisms (e.g., crack deflection, crack bridging, and microcracking). Residual stresses could influence the capability of materials to absorb energy from external loading and distribute damage, consequently leading to enhanced quasi-plasticity and flaw tolerance. The occurrence of spontaneous microcracking in AT was also reported to be caused by residual stresses induced by the strong thermal expansion anisotropy of AT ($\alpha_{a,AT25-1000\;{}^{\circ}C}$ = −2.4 × 10^−6^ °C^−1^, $\alpha_{b,AT25-1000\;{}^{\circ}C}$ = 11.9 × 10^−6^ °C^−1^ and $\alpha_{c,AT25-1000\;{}^{\circ}C}$= 20.8 × 10^−6^ °C^−1^) [13], resulting in a low elastic modulus, approximately 25–40 GPa [14]. In contrast, in Botero’s study [9], a significantly improved elastic modulus was attained in fine-grained AT using nanoindentation in dense A–AT composites without microcracks; this was attributed to the residual stress interaction between matrix and reinforcing particles. These statements show that the mechanical properties of A–AT composites are considerably related to residual stresses and microstructure. To improve A–AT ceramic properties, the strict control of the microstructure and clear comprehension of the nature as well as the magnitude of residual stresses generated during fabrication are necessary.

The interest in developing laminated ceramics to achieve superior flaw tolerance is growing [15]. In addition to the effect of laminate stacking design (e.g., layer thickness and stacking structure), detailed residual stress information (e.g., residual stresses between phases and grains) regarding each layer is crucial. This is extremely important to understand the reinforcing mechanism and optimize the resulting residual stress field, which is useful to produce optimum laminated structures with operative reinforcing layers. It was supposed that the residual stresses in ceramics could be modified by selecting the appropriate material design and fabrication process [16,17]. For A–AT ceramics, the addition of AT and the implementation of sintering treatments can considerably influence their microstructure and mechanical properties [18,19]. Because the co-sintering of layers with different compositions in laminated materials is required, the effects of introducing AT and sintering treatments on residual stress development in addition to those related to laminate stacking design must be considered.

Several studies on the residual stress analysis of A–AT composites have been conducted. Singh [20] and Skala et al. [7] used synchrotron radiation diffraction and X-ray diffraction (XRD) techniques in their investigation. They employed single-peak shift analysis for residual strain measurement in functionally graded A–AT composites. However, they only considered the shifts of individual peaks of Al_2_O_3_ and AT; residual strain was presented without residual stresses. Fluorescence piezo-spectroscopy was implemented to detect stress in each layer of the multilayered Al_2_O_3_–Al_2_TiO_5_ composites. The results indicated weak tension in AT layers (5–20 MPa) and compressive hydrostatic stresses in alumina layers (20 MPa) [10]. The residual stresses in each A–AT laminate layer were also evaluated using simplified model calculations [21]. The reported tensile stresses in A-10AT (composite containing 10 vol.% AT) and compressive stresses in A-30AT (containing 30 vol.% AT) layers were expected to be approximately 15 and 90 MPa, respectively. According to previous work, the residual stress results were presented in different values, depending on fabrication routes, composition design, and residual stress determination techniques. Most studies focus on uniform residual stresses in between layers, laminates, or phases. However, they do not account for thermal and elastic crystal anisotropies at the grain-scale level, which may perform a significant function in crack initiation processes, such as fatigue or failure. Furthermore, owing to crystal anisotropy and microstructure complexity, the residual strains from single-peak diffraction measurements or model calculations may not represent bulk material behavior [22]. To date, direct and reliable experimental data on multi-scale residual stress in A–AT composites remain lacking. To accurately quantify residual stresses under different fabrication conditions or material compositions, the most suitable residual stress measurement technique for each case may be implemented.

The time-of-flight (TOF) neutron diffraction technique allows for the non-destructive measurement of residual strains in bulk materials because it is capable of high neutron penetration. In addition, the entire diffraction pattern, which is important for determining the residual stress in complex materials, such as ceramic composites, can be determined [23]. Information on multi-scale residual stresses, such as average stress among phases, intergranular stress state within a single phase, and even nonuniform microstrains at the atomic scale, can be derived from the analysis of the entire diffraction pattern [22].

In this study, TOF neutron diffraction and Rietveld analysis were applied to precisely determine the residual stresses in a series of A–AT composites with different AT contents and sintering treatments and, therefore, with significantly different microstructures. Multi-phase qualitative and quantitative analyses as well as crystal structure determination were performed using Rietveld refinement. The through-thickness residual stress profiles of the mean phase stress and intergranular stress state of each phase were obtained for all the examined A–AT composites. The effects of second-phase AT addition and sintering temperature were discussed based on the observed microstructures and detected residual stresses.

## 2. Experimental

### 2.1. Sample Preparation and Characterization

High-purity α-Al_2_O_3_ (Condea HPA 0.5, USA; *d*_50_ = 0.35 µm) and TiO_2_ (anatase; Merck, 808, Darmstadt, Germany; *d*_50_ = 0.35 µm) powders were used as starting powders. Monoliths of Al_2_O_3_–Al_2_TiO_5_ (A–AT) composites with 10 and 40 vol.% of AT were manufactured by the reaction sintering of green compacts prepared from mixtures of Al_2_O_3_ and TiO_2_ with relative TiO_2_ contents of 5 and 20 wt.%, respectively. Following colloidal filtration techniques [24], the powder mixtures were dispersed in deionized water by adding 0.5 wt.% (on a dry solids basis) of a carbonic acid-based polyelectrolyte (Dolapix CE64, Zschimmer-Schwarz, Lahnstein, Germany). Stable suspensions of a mixture of undoped alumina and titania with a solid loading of 80 wt.% were prepared by 4 h ball milling, using an alumina jar and balls. Green compact blocks, 70 mm × 70 mm with 12 mm thickness, were slip cast in plaster of Paris molds. The cast bodies were carefully removed from the molds and dried in air at room temperature for a minimum of 24 h. The dried blocks were sintered in an electrical box furnace (Termiber, Madrid, Spain) with heating and cooling rates of 2 °C/min. The first step involved sintering at 1200 °C with a 4 h dwell time during heating. In the second step, two different treatments with different maximum sintering temperatures, i.e., 2 h dwell times at 1450 and 1550 °C, were implemented. The materials were denoted as A-10AT(1450), A-10AT(1550), A-40AT(1450), and A-40AT(1550) to describe compositions and sintering temperatures.

The phase identification of the sintered specimens was performed using an X-ray diffractometer (XRD, DX-2700, Dandong, China) using CuKα radiation at room temperature over a 2θ range of 10 to 80°, with a step size of 0.03° and a counting time of 1.0 s at each step. Quantitative phase analysis was undertaken using the Rietveld pattern fitting method. Microstructural characterization of polished and chemically etched (HF 10 vol.%, 1 min) samples was performed by scanning electron microscopy (SEM). The average grain sizes of Al_2_O_3_ and AT particles were determined using the linear intercept method, considering at least 200 grains for each phase. The densities of samples were determined using Archimedes’ method (European Standard EN 1389:2003). The relative densities of α-Al_2_O_3_ (ASTM 42-1468) and AT (ASTM 26-0040) were calculated as percentages of their theoretical densities, i.e., 3.99 and 3.70 g/cm^3^, respectively. The flexural strength was determined by a three-point bending test on the rectangular bar samples (geometry of 28 × 10 × 2 mm^3^), under conditions of a span length of 20 mm and a constant loading speed of 0.5 mm/min. For each material, three samples were prepared for the bending test to obtain an average value and the standard deviation. The fracture surfaces of the fractured samples were characterized by SEM after the three-point bending test.

### 2.2. Residual Stress Measurement

**Figure 1:**
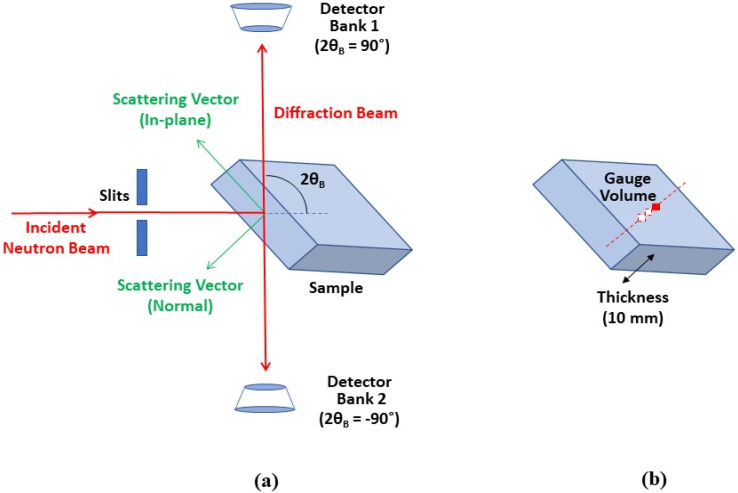
Experimental setup on TOF neutron strain scanner ENGIN-X at ISIS. (**a**) Residual strains were collected both in in-plane and normal directions. (**b**) Strain scanning was implemented along sample thickness with interior gauge volume.

For neutron diffraction strain scanning, rectangular parallelepiped samples (30 × 30 × 10 mm^3^) were employed. Residual strain measurements were performed by neutron diffraction on the ENGIN-X TOF instrument [25] at the ISIS neutron and muon source in Rutherford Laboratory, UK. Two detector banks were set at Bragg angles of 2$\theta_{B}$ = ±90° for simultaneous strain measurement in two directions: the in-plane direction (parallel to the major plane of 30 mm × 30 mm samples) and normal direction (perpendicular to the major plane of 30 mm × 30 mm samples). The experimental setup is illustrated in Figure 1.

The set measurement gauge volume was 16 × 3 × 1 mm^3^, with the centroid defined as the location of measurement. Through-thickness strain scanning was implemented along the sample thickness (10 mm) with a 1.5 mm measurement step. To avoid anomalous strain due to the effects of partial neutron gauge volume filling near the surface [26], the scanning points were no closer than 2 mm from the sample surfaces. The stress-free reference lattice parameters of Al_2_O_3_ and AT phases were obtained by measuring Al_2_O_3_ and AT powders with the same gauge volume. As the experimental standard, CeO_2_ powder was measured to calibrate the peak profile parameters to be employed in Rietveld refinement. All the measurements were performed at room temperature.

### 2.3. Data Analysis

The entire diffraction spectrum was obtained from TOF diffraction measurements. Diffraction spectra were analyzed by the Rietveld refinement of the entire spectrum and by the single-peak fitting of each hkl reflection using the TOPAS-Academic V5 software package [27].

#### 2.3.1. Rietveld Refinement

Rietveld refinement is a well-known structure refinement method of the entire profile based on least-squares fitting. It is powerful for determining the complete crystal structure (e.g., lattice parameters, atomic coordinates, and occupancy) of a multiphase material [28,29]. The peak position, intensity, and width of each phase were independently derived. Quantitative phase analysis was also performed using Rietveld analysis.

The ENGIN-X instrument diffraction profile was modeled using a convolution of a pseudo-Voigt function, pV(t), with two back-to-back exponentials, E(t) [22]. Instrument-dependent diffractometer constants and profile parameters were calibrated by the refinement of data from the CeO_2_ standard powder (cubic phase, space group $Fm\bar{3}m$, and a = 5.4114 Å). The derived parameters were then used to refine the studied bulk samples and stress-free reference powders.

The refinements of A–AT composites were undertaken with a two-phase model consisting of a hexagonal α-Al_2_O_3_ phase and an orthorhombic AT phase. Parameters, such as lattice parameters, atomic coordinates, isotropic thermal parameters, scale factors, and polynomial background parameters, were improved by Rietveld refinement. All refinements were implemented step-by-step to avoid correlation effects among parameters. The space groups and initial atomic structure information used in the refinements are listed in Table 1. The overall fitting quality was assessed in terms of R values [30] obtained from the refinements.

#### 2.3.2. Stress Determination

Based on the entire diffraction spectrum, residual stresses can be calculated either from the change in lattice parameters of each phase or from individual hkl peak shifts. The former represents the average stress behavior of each phase (called mean phase stress), and the latter represents the stress in individual grains (called peak-specific residual stress).

#### Mean Phase Stresses

The mean phase strain, given by the weighted average of several single-peak strains, was determined from the change in the average lattice parameters of each phase in composites with respect to those in stress-free reference powders [28]. In this case, all diffraction peaks were considered, thus allowing the representation of residual stresses more precisely and reliably for bulk composites.

In this study, the strain for the hexagonal *α*-Al_2_O_3_ phase can be calculated along lattice axes *a* and *c*:

(1) $$\varepsilon_{a}=\frac{a-a_{0}}{a_{0}}$$

(2) $$\varepsilon_{c}=\frac{c-c_{0}}{c_{0}}$$

where a (a=b) and c are the lattice parameters of the *α*-Al_2_O_3_ phase, and $a_{0}$ and $c_{0}$ are the stress-free lattice parameters measured from the Al_2_O_3_ starting powder. For orthorhombic AT, the strains in the a, b, and c axes can be similarly determined.

Without considering granular anisotropy, the mean phase strains of the Al_2_O_3_ and AT phases in gauge volume were calculated by averaging the strain over the unit cell, as presented in [32].

(3) $${\bar{\varepsilon }}_{A}=\frac{1}{3}{\left(2\varepsilon_{a}+\varepsilon_{c}\right)}_{A}$$

(4) $${\bar{\varepsilon }}_{AT}=\frac{1}{3}{\left(\varepsilon_{a}+\varepsilon_{b}+\varepsilon_{c}\right)}_{AT}$$

Considering the fabrication process and geometrical symmetry of samples, the strains were assumed to be isotropic in a direction parallel to the major plane of samples. Thus, the strains measured in the in-plane and normal directions are sufficient for stress determination. These are used as principal strains $\varepsilon_{ii}$ (i = 1, 2, 3), $\varepsilon_{11}=\varepsilon_{22}=\varepsilon_{In-plane}$, and $\varepsilon_{33}=\varepsilon_{Normal}$. Thus, the mean phase stresses of Al_2_O_3_ and AT were determined in the in-plane and normal directions using Hooke’s law, respectively, as follows:

(5) $$\sigma_{In-plane}=\frac{E}{1+\nu }{\bar{\varepsilon }}_{In-plane}+\frac{E\nu }{\left(1+\nu \right)\left(1-2\nu \right)}\left(2{\bar{\varepsilon }}_{In-plane}+{\bar{\varepsilon }}_{Normal}\right)$$

(6) $$\sigma_{Normal}=\frac{E}{1+\nu }{\bar{\varepsilon }}_{Normal}+\frac{E\nu }{\left(1+\nu \right)\left(1-2\nu \right)}\left(2{\bar{\varepsilon }}_{In-plane}+{\bar{\varepsilon }}_{Normal}\right)$$

where $\bar{\varepsilon }$ corresponds to the calculated mean phase strains of Al_2_O_3_ and AT phases, as given by Equations (3) and (4), respectively; and E and ν are the bulk elastic constants of Al_2_O_3_ (E = 400 GPa and ν = 0.22) [33] and AT (E = 284 GPa and ν = 0.33) [9].

#### Peak-Specific Residual Stresses

Owing to single-crystal anisotropy, the strain measured by diffraction varies among grain families; consequently, such is the case among diffraction peaks [34]. The deviation from the average phase stress in a given grain, that is, the peak-specific residual stresses, can be obtained by measuring the shifts in individual diffraction peaks. Understanding peak-specific residual stresses is important because the stress concentrations at the scale of individual grains may ultimately affect crack initiation processes in fatigue or brittle failure.

In TOF diffraction, at a fixed scattering angle, 2$\theta_{B}$, the lattice spacing, $d_{hkl}$, of the hkl plane can be obtained from the TOF of the peak position, $t_{hkl}$ [23]:

(7) $$d_{hkl}=\frac{h}{2\sin\theta_{B}mL}t_{hkl}$$

where h is the Planck constant, m is the neutron mass, and L is the neutron flight path length.

The peak-specific residual strain, which depends on the change in the lattice spacing of the $\Delta d_{hkl}$ hkl plane, can then be calculated in terms of the TOF shift in the recorded peak, $\Delta t_{hkl}$:

(8) $$\varepsilon_{hkl}=\Delta d_{hkl}/d_{hkl}^{0}=\Delta t_{hkl}/t_{hkl}^{0}$$

where $d_{hkl}^{0}$ is the strain-free reference lattice spacing, $t_{hkl}^{0}$, and strain-free reference TOF at the hkl peak. The peak positions can be precisely determined by single-peak fitting, with a typical sensitivity of $\delta_{\varepsilon }=\Delta d_{hkl}/d_{hkl}≅50\cdot 10^{-6}\;$. The peak-specific residual strains measured along the in-plane and normal directions were characterized as $\varepsilon_{In-plane}^{hkl}$ and $\varepsilon_{Normal}^{hkl}$, respectively. They were considered as principal strain components $\varepsilon_{ii}$ (i = 1, 2, 3; $\varepsilon_{11}=\varepsilon_{22}=\varepsilon_{In-plane}$; and $\varepsilon_{33}=\varepsilon_{Normal}$).

The peak-specific residual stresses for each reflection of Al_2_O_3_ and AT in the in-plane and normal directions ($\sigma_{In-plane}^{hkl}$ and $\sigma_{Normal}^{hkl}$) were calculated using Hooke’s law, as follows:

(9) $$\sigma_{In-plane}^{hkl}=\frac{E_{hkl}}{1+\nu_{hkl}}\varepsilon_{In-plane}^{hkl}+\frac{E_{hkl}\nu_{hkl}}{\left(1+\nu_{hkl}\right)\left(1-2\nu_{hkl}\right)}\left(2\varepsilon_{In-plane}^{hkl}+\varepsilon_{Normal}^{hkl}\right)$$

(10) $$\sigma_{Normal}^{hkl}=\frac{E_{hkl}}{1+\nu_{hkl}}\varepsilon_{Normal}^{hkl}+\frac{E_{hkl}\nu_{hkl}}{\left(1+\nu_{hkl}\right)\left(1-2\nu_{hkl}\right)}\left(2\varepsilon_{In-plane}^{hkl}+\varepsilon_{Normal}^{hkl}\right)$$

where $E_{hkl}$ and $v_{hkl}$ are the diffraction elastic constants (DECs) corresponding to the hkl reflection in each corresponding phase. In the present work, the DECs of Al_2_O_3_ (hkl) and AT (hkl) were obtained using the program IsoDEC [35,36], following the Kröner model.

## 3. Results and Discussions

### 3.1. Phase Composition and Microstructure

**Figure 2:**
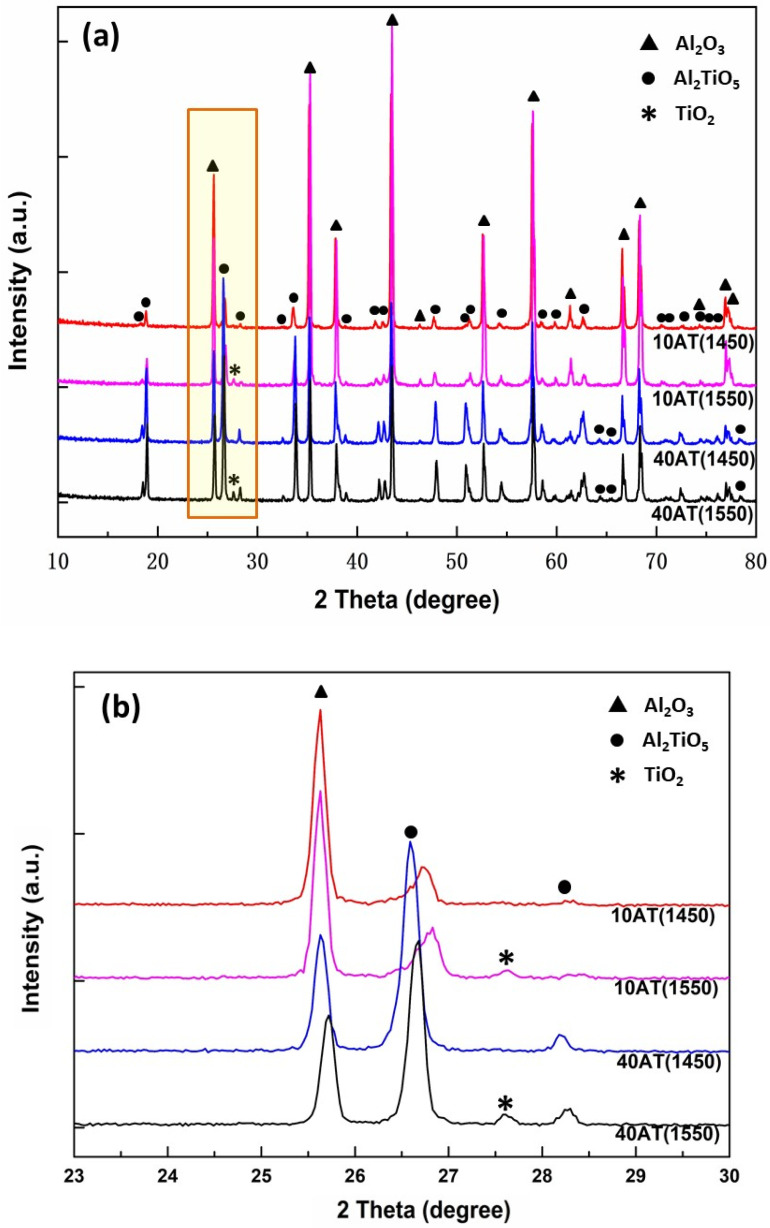
XRD patterns of studied Al_2_O_3_–Al_2_TiO_5_ bulk ceramic composites. (**a**) The full XRD patterns at the range of 2θ = 10~80° and (**b**) partial enhancement at the range of 2θ = 23~30°, as marked in (**a**) with a yellow rectangle.

The XRD profiles of the studied A–AT composites are shown in Figure 2.

In the A-10AT(1450) and A-40AT(1450) composites, only peaks corresponding to the Al_2_O_3_ and AT phases were detected without the TiO_2_ phase. This demonstrates that the initial titania powders completely reacted with alumina and transformed into AT during fabrication. A previous study [37] investigated the dynamic phase formation in the temperature range of 20–1400 °C for the fabrication of A–AT samples by means of neutron diffraction and differential thermal analysis. The results showed that the formation of AT in A–AT samples occurred at temperatures exceeding 1310 °C by the reaction sintering of the Al_2_O_3_ and TiO_2_ mixture; TiO_2_ disappeared at 1370 °C. Violini et al. [38] reported that in the two-step reaction sintering of initial powders (alumina and titania), the formation of AT started at 1380 and ended at 1440 °C. In our work, the sintering temperature 1450 °C ensured the complete formation of AT. However, in the XRD profiles of the A-10AT(1550) and A-40AT(1550) composites, in addition to the main peaks of the Al_2_O_3_ and AT phases, a small peak of TiO_2_ (rutile) phase was detected at around 2θ = 7.6° (hkl = 110). This indicated the existence of a TiO_2_ (rutile) phase in the 1550 °C sintered A–AT composites. The results of quantitative phase analysis demonstrated that the content of TiO_2_ was very limited, with values of 0.5 wt.% and 1.2 wt.% in the A-10AT(1550) and A-40AT(1550) composites, respectively.

According to the previous discussion, the complete reaction from initial titania and alumina to AT can be guaranteed with a sintering temperature higher than 1450 °C. The detected TiO_2_ phase in these 1550 °C sintered A–AT composites could be formed due to a partial decomposition of the AT phase during cooling. It is well known that pure synthetized AT is thermally unstable in the temperature range of 800–1280 °C. It tends to decompose, through a eutectoid reaction, into α-Al_2_O_3_ and TiO_2_ (rutile) during cooling from sintering treatments [13,39]. This brings disadvantage for the material, such as reduced thermal shock resistance. It is well accepted that the decomposition of AT is a nucleation-and-growth process. Experimental evidence has suggested that AT can be thermally stabilized by limiting its grain growth [40,41]. The heat treatment temperature plays an important role in the grain growth progress. In our study, with a smaller AT grain size in 1450 °C sintered A–AT materials, compared with the one in 1550 °C sintered samples, the AT phase exhibited increased thermal stability, i.e., without decomposition.

**Figure 3:**
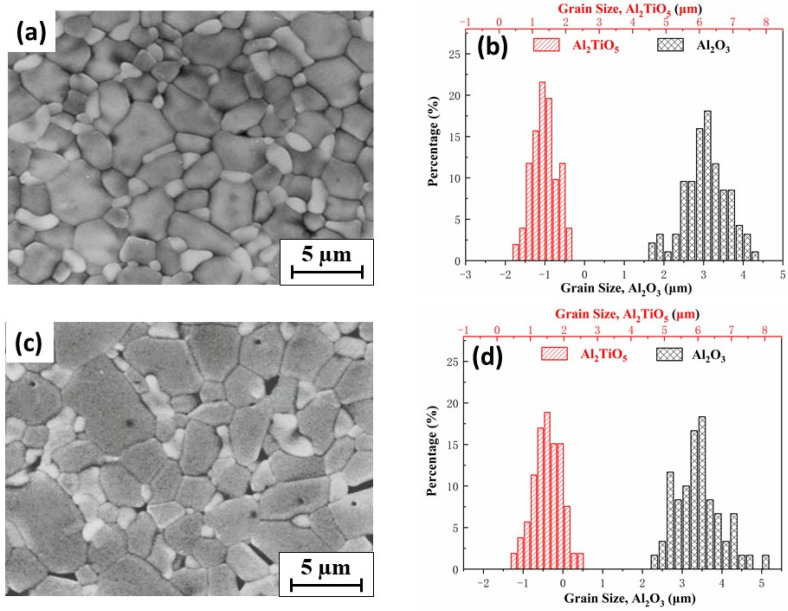

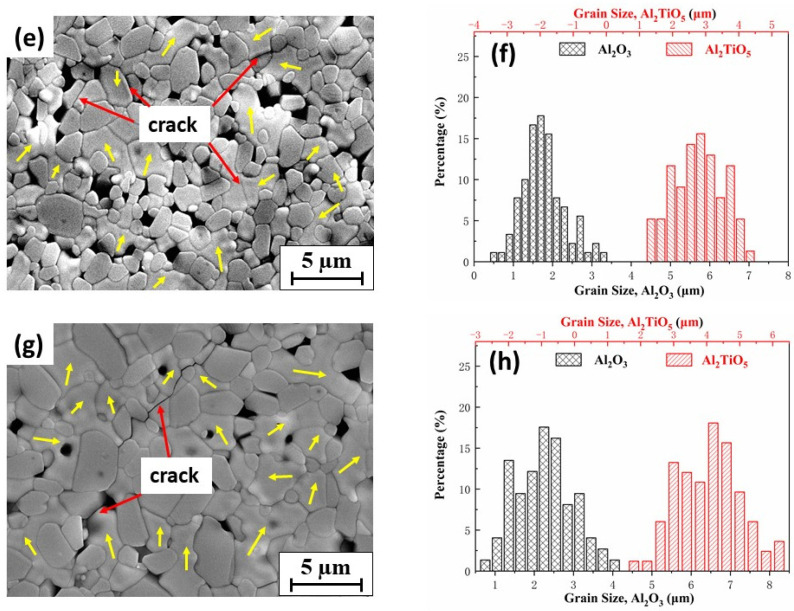
SEM images of polished and etched surfaces of Al_2_O_3_–Al_2_TiO_5_ samples and size distribution of Al_2_O_3_ and Al_2_TiO_5_ particles in each sample: (**a**,**b**) A-10AT(1450); (**c**,**d**) A-10AT(1550); (**e**,**f**) A-40AT(1450); and (**g**,**h**) A-40AT(1550). Yellow arrows indicate locations of AT phases in A-40AT composites.

The characteristic microstructures of the investigated A–AT composites are shown in Figure 3.

A dense microstructure was observed in the A-10AT composites (Figure 3a,c) irrespective of the sintering temperature (i.e., 1450 and 1550 °C). In the back-scattered electron images, AT grains and alumina matrix appeared with light and dark gray colors, respectively. Round-shaped and slightly elongated-shaped AT grains (1.3–1.6 μm) were homogeneously distributed and mainly located at the triple points and grain boundaries of the alumina matrix. A relatively narrow distribution of grain sizes for both the Al_2_O_3_ matrix and second-phase AT was observed in the A-10AT composites, according to the histogram of grain size distribution in Figure 3b,d. As the AT content increased to 40 vol.%, the grain size of Al_2_O_3_ decreased, whereas the AT grain size increased (Table 2). The AT grains in the A-40AT composites exhibited a distinct irregular shape and heterogeneity in size distribution, as indicated by the arrows in Figure 3e,g. Some AT grains clustered together as a submatrix (4–6 μm), where alumina grains were separated and surrounded by AT grains; a similar phenomenon was also reported in another study [38]. The Al_2_O_3_ grain size evidently decreased as the AT content increased owing to the inhibiting effect of second-phase AT particles on the Al_2_O_3_ matrix grain growth. This phenomenon was also observed in other alumina-based ceramics, such as alumina–zirconia ceramics [22,42]. As the AT content increased, more pores and microcracks were observed in their SEM images. This corresponds to the reduced density tendency summarized in Table 2.

**Figure 4:**
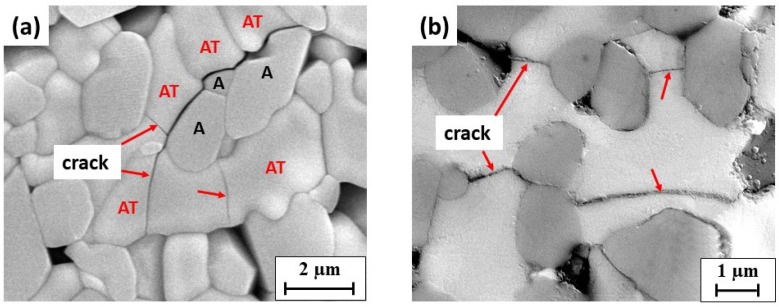
SEM micrograph showing microcracks in A-40AT samples: (**a**) A-40AT(1450). The aluminum titanate and alumina grains are marked as “AT” and “A”, respectively; (**b**) BSE-SEM image of A-40AT(1550). Alumina grains in dark gray, and aluminum titanate in light gray. Red arrows indicate locations of microcracks.

Figure 4 exhibits that microcracks were mainly along the grain boundaries between Al_2_O_3_ and AT; some transgranular cracks, acting as bridges between one alumina grain to another, were present in the AT grains. Such microcracks propagation behaviors reflect the complex stresses in A–AT composites during fabrication.

The effect of sintering temperature was observed as follows. At higher sintering temperatures with the same composition, the studied A–AT composites presented a coarser microstructure associated with the grain growth of Al_2_O_3_ and AT. This phenomenon is particularly evident in the A-40AT composites. In these composites, at a higher sintering temperature (i.e., 1550 °C), the excessive grain growth of Al_2_O_3_ and AT was promoted after the formation of the AT phase. Owing to the crystallographic anisotropy of thermal expansion in the orthorhombic structure, the grain growth of AT particulates became more abnormal. Microcracks form once the grain size of AT reaches the critical size (approximately 2 μm) necessary for microcracking [38,43]. Compared with the A-40AT(1450) composites, more microcracks appeared in the A-40AT(1550) composites.

**Figure 5:**
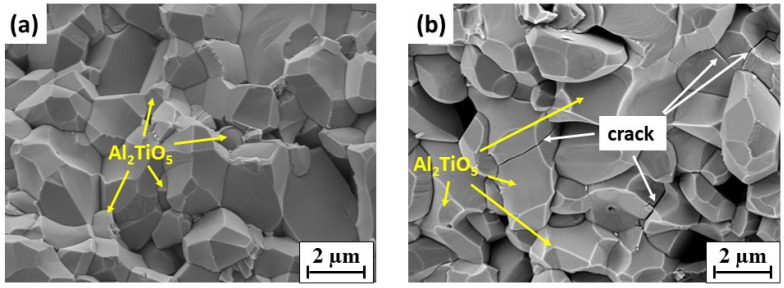
SEM images of fracture surfaces of Al_2_O_3_–Al_2_TiO_5_ composites. Arrows indicate Al_2_TiO_5_ particles and microcracks. (**a**) A-10AT composites irrespective of sintering temperature and (**b**) A-40AT composites irrespective of sintering temperature.

These microcracks function as excellent regions for main crack energy absorption and dissipation, leading to remarkable crack attenuation and deflection. Such a mechanism can be confirmed by the fracture surface morphologies of A–AT bulk ceramic composites (Figure 5).

Without microcracking, the predominant fracture mode of A-10AT composites (Figure 5a) was intergranular fracture. In contrast, with weaker grain boundaries and microcracks in the A-40AT composites (Figure 5b), transgranular fracture appeared in AT particles in addition to the intergranular fracture along weak boundaries between particles. The existence of these microcracks and “weak” grain boundaries is presumed to impart low mechanical strength; however, flaw tolerance, such as high thermal shock resistance and improved thermal stability, improves [44]. This point corresponds well with the results of flexure strength of the studied A–AT composites, as given in Table 2. With the increase of AT content, the flexure strength of A–AT composites remarkably decreased, mainly due to the existence of microcracks. As the sintering temperature increased from 1450 to 1550 °C, the flexure strength slightly reduced, which was related to the coarser microstructure and lower density of the composites.

Attempts were made to identify the existence of TiO_2_ in the microstructure of 1550 °C sintered A–AT composites. However, no significant evidence of TiO_2_ was detected in the SEM micrographs of the A-10AT(1550) and A-40AT(1550) composites, owning to the insufficient content of TiO_2_.

### 3.2. Neutron Diffraction Patterns and Rietveld Analysis

**Figure 6:**
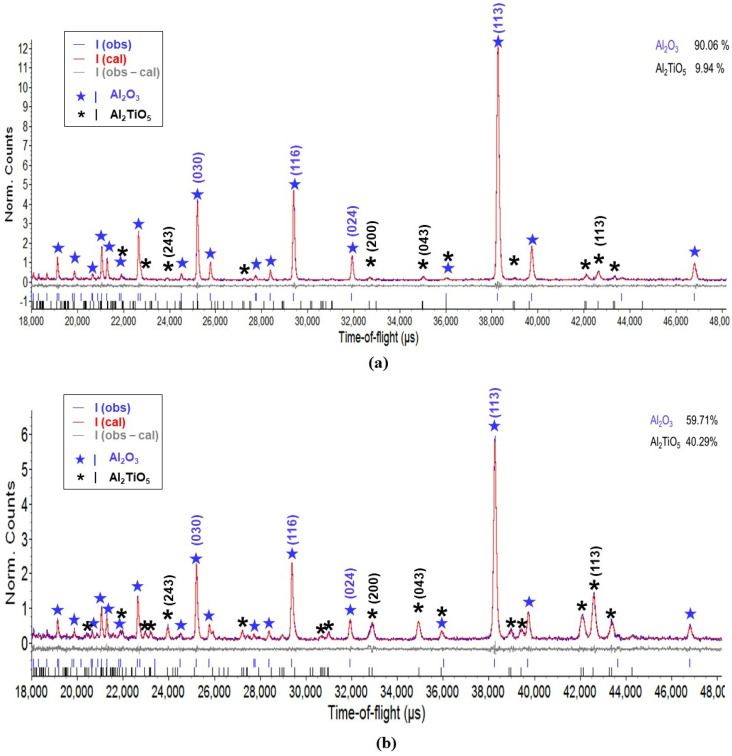
Representative TOF neutron diffraction patterns analyzed by Rietveld method for (**a**) A-10AT(1550) and (**b**) A-40AT(1550) samples. Investigated reflections are marked above peaks with ★ for Al_2_O_3_ and * for Al_2_TiO_5_.

No distinct differences were observed among the TOF diffraction patterns of A–AT samples with the same AT contents at various sintering temperatures; the diffraction spectra mainly varied with the AT content. The representative diffraction patterns of the A-10AT(1550) and A-40AT(1550) composites are shown in Figure 6. As the AT content increased, the peak intensities of the Al_2_O_3_ matrix decreased; this is in contrast to the increase in AT intensity. In addition, at the same scanning point, the differences between the spectra in the in-plane and normal directions (measured at 2θ = ±90°) are insignificant.

In Figure 6, raw data collected by the diffraction method (observed) are represented by a blue line, and data derived by Rietveld refinement are denoted by a red line overlapping with the blue (observed) pattern. Unlike the results in the XRD analysis, only peaks of the Al_2_O_3_ and AT phases without evidence of the TiO_2_ phase are observed in the TOF diffraction patterns of A-AT(1550) samples, which is concerned with the limited content of TiO_2_. Due to the difficulty in identifying TiO_2_ in the microstructure and neutron diffraction analysis, it can be considered that the effect of TiO_2_ on residual stresses of the composites would be extremely limited, and thus the crystal structure and stress analysis of TiO_2_ were not involved in the following results and discussion. The Rietveld refinement of a two-phase model consisting of α-Al_2_O_3_ and AT phases was highly satisfactory for all A–AT bulk samples. The positions of individual peaks of α-Al_2_O_3_ and AT phases were identified with blue and black tick marks at the bottom of the pattern, respectively. The gray line below the pattern represents the difference between the observed and calculated intensities, which were virtually flat in both the A-10AT and A-40AT composites. This indicates the excellent fits in Rietveld refinement that were achieved for all the reference powders and A–AT bulk samples; the weighted residual error, $R_{wp}$, ranged from 3 to 7%.

The phase composition corresponding to the measured gauge volumes was evaluated by Rietveld refinement; the volume fraction is shown on the upper right of the diffraction pattern fitting window (Figure 6). Virtually constant AT contents were recorded at different scanning positions of the same sample. The calculated AT volume fractions were 9.5 ± 1 and 40 ± 2 vol.% in the A-10AT and A-40AT composites, respectively; these results agreed well with the nominal values.

### 3.3. Crystal Structures

After applying Rietveld refinement to TOF neutron diffraction patterns, the crystal structures of Al_2_O_3_ and AT in the stress-free reference powders and A–AT composites were obtained. Table 3 summarizes the average values of refined lattice parameters of α-Al_2_O_3_ (a, c) and orthorhombic AT (a, b, and c) at different scanning points in each sample (standard deviations are shown in brackets). Based on these standard deviations, no remarkable variance was found in the lattice parameters of both Al_2_O_3_ and AT in the in-plane and normal directions as well as at different scanning points in the same bulk sample. This indicates the existence of phases and microstructure distributions that are virtually homogeneous in the above materials.

The lattice parameters (a and c) of Al_2_O_3_ in all the studied A–AT ceramics were found to be slightly lower than those of the initial Al_2_O_3_ powders. Thus, compressive strain was anticipated in the Al_2_O_3_ phase of A–AT ceramics. With increasing AT content, changes in the lattice parameters of Al_2_O_3_ were not remarkable, and the c value only slightly decreased. Compared with the initial AT powders, the a value of orthorhombic AT in all the studied A–AT ceramics decreased, whereas the b and c values increased. As the AT content increased from 10 to 40 vol.%, the a value of AT increased, whereas the b and c values distinctly decreased.

In comparing the A–AT samples with the same composition but fabricated at different sintering temperatures, no remarkable difference was observed among the lattice parameters of the Al_2_O_3_ and AT crystallites, except for AT in the A-40AT samples. In the A-40AT composites, the materials sintered at a higher temperature value (1550 °C) exhibited an increase in the a values of the AT phase; however, the b and c values reduced compared with the materials sintered at 1450 °C.

These behaviors can be understood by considering the thermal expansion mismatch between the Al_2_O_3_ and AT phases, the thermal expansion anisotropy of each phase, and the microstructure characteristics of composites. According to the microstructure described above, the A-10AT composites (Figure 3a,b) were dense, and fine AT grains were surrounded by Al_2_O_3_ matrix grains. The average crystallographic thermal expansion (CTE) coefficient of the Al_2_O_3_ matrix ($\alpha_{A,25-1000\;{}^{\circ}C}$ = 8.6 × 10^−6^ °C^−^^1^) is smaller than that of the AT inclusions ($\alpha_{AT,25-1000\;{}^{\circ}C}$ = 10.1 × 10^−6^ °C^−^^1^) [24] when subjected to cooling from the maximum sintering temperature to room temperature in the fabrication procedure. Consequently, Al_2_O_3_ matrix grains were compressed, and lattice parameters a and c decreased.

In contrast, for AT particles, most of the contact and restraints emanated from the Al_2_O_3_ matrix in the A-10AT composites. Owing to the CTE mismatch between Al_2_O_3_ and AT, the AT grains were presumed to be in tension, and the lattice parameters increased. However, according to the lattice parameters obtained by neutron diffraction, the b and c values of orthorhombic AT in A-10AT ceramics increased as the a value decreased. The strong CTE anisotropy exhibited by AT ($\alpha_{a,AT25-1000\;{}^{\circ}C}$ = −2.4 × 10^−6^ °C^−1^, $\alpha_{b,AT25-1000\;{}^{\circ}C}$ = 11.9 × 10^−6^ °C^−1^ and $\alpha_{c,AT25-1000\;{}^{\circ}C}$= 20.8 × 10^−6^ °C^−1^) [13] indicates that this anisotropy is the predominant effect rather than the CTE mismatch with Al_2_O_3_, although only a small quantity of AT was included in the A–AT ceramics. By increasing the AT content, the contact among AT grains was enhanced. Most restraints were from the closed AT grains, and the effect of CTE anisotropy on the AT phase became more distinct. A decrease in the a value and further increases in b and c values were anticipated in AT. However, the derived lattice parameter results compared with those in the A-10AT ceramics showed that the a value of AT increased in the A-40AT ceramics, whereas the b and c values decreased. Such phenomena can be explained by the microstructure of the A–AT composites. As presented in Figure 3, spontaneous microcracking occurred in the AT of the A-40AT composites. Thus, restraints among grains were evidently released, and changes in the lattice parameters of AT weakened. This also explains the difference in AT lattice parameters between the A-40AT(1450) and A-40AT(1550) ceramics. At higher sintering temperatures, more microcracks were observed in A-40AT(1550) than in A-40AT(1450). Thus, changes in the lattice parameters of AT in the A-40AT(1550) ceramics compared with the A-40AT(1450) materials weakened, leading to an increase in a values of the AT phase but reductions in the b and c values.

In addition, the lattice parameters could reflect the thermal stability of Al_2_TiO_5_. Skala et al. [13] reported that the thermal stability of Al_2_TiO_5_ is closely related to its lattice constant c. Its increase will lead to a reduction of the distortion of the octahedra in the crystal structure of Al_2_TiO_5_, so that the stability of Al_2_TiO_5_ is improved. In our study, in A–AT ceramics with the same AT addition, as the sintering temperature increased from 1450 °C to 1550 °C, the lattice parameter c of AT reduced, indicating less thermal stability of the AT phase. This explains why the decomposition of AT occurred in the 1550 °C sintered A–AT samples.

### 3.4. Stress Determination

#### 3.4.1. Mean Phase Stresses

**Figure 7:**
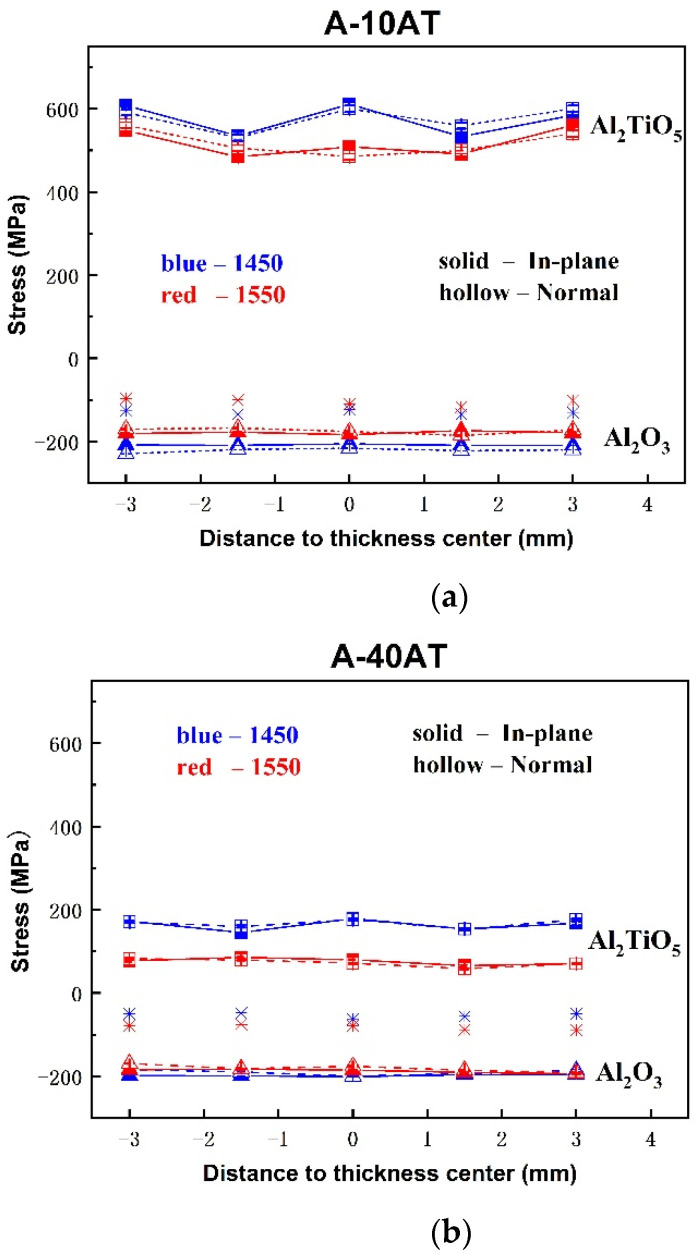
Mean phase stress profiles for Al_2_O_3_ (Δ) and Al_2_TiO_5_ (□) phases; through-thickness of Al_2_O_3_–Al_2_TiO_5_ bulk samples as a function of Al_2_TiO_5_ contents and sintering temperatures (1450 °C in blue line and 1550 °C in red line); values of $f_{A}\sigma_{A}+f_{AT}\sigma_{AT}$ are presented (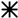) without a line. (**a**) A-10AT composites and (**b**) A-40AT composites.

Mean phase stress, the average stress in each phase over several randomly oriented grains within the gauge volume area in the TOF measurement, was calculated using the change in the lattice parameters of the Al_2_O_3_ and AT phases, as given by Equations (1)–(6), respectively. The through-thickness mean phase stress profiles measured in the in-plane and normal directions are depicted in Figure 7 for the A–AT bulk samples with different AT additions and sintering temperatures (1450 and 1550 °C). Note that error bars corresponding to the statistical uncertainties of the determined lattice parameters are smaller than the size of the symbols employed in the presented graphs.

As shown in Figure 7, the mean phase stress behaviors between the normal and in-plane directions are similar in all cases. The through-thickness stress profiles of both AT and Al_2_O_3_ phases are virtually flat, with residual tensile stresses in AT particulates and compressive stresses in the Al_2_O_3_ matrix. The high tensile stresses in AT were approximately 500–610 MPa for the A-10AT composites irrespective of the sintering temperature. It rapidly decreased as the AT content increased to approximately 80–180 MPa in the A-40AT composites. As the AT vol.% changed, the compressive stresses in the Al_2_O_3_ did not vary and remained at approximately −200 ± 30 MPa.

In addition, the residual stress behaviors according to different sintering treatments were studied. Regarding A–AT ceramics with the same AT addition, as the sintering temperature increased from 1450 to 1550 °C, tension in the AT phase decreased, and absolute compression values in the Al_2_O_3_ matrix slightly decreased in the A-10AT composites; however, no distinct variations were found in the A-40AT composites.

The residual thermal stresses in a particulate-reinforced composite are known to be caused by the elastic deformations of the matrix and particulates under uniform temperature change [45]. This is mainly due to the mismatch of thermal expansions and elastic constants between the matrix and particulates. For A–AT composites, the average crystallographic thermal expansion coefficient for the Al_2_O_3_ matrix is smaller than that for the AT inclusion [13,46,47]. Therefore, during fabrication, which is subjected to cooling from the maximum sintering temperature to room temperature, high compressive residual thermal stresses were induced in the Al_2_O_3_ matrix and tension in the AT inclusion.

Microstructural factors, such as particle volume fraction, size, shape, and microcracking, are known to also affect the magnitude and distribution of residual stresses. Considering the microstructure and grain size of AT and Al_2_O_3_ in A–AT samples with high AT contents, the grain size of AT was observed to increase. For a single AT particle, the surrounding misfit effects weakened because of the reduction in the contact area with the Al_2_O_3_ matrix and the increase in the contact area among AT particulates. The tension produced in AT due to the CTE misfit with the Al_2_O_3_ matrix correspondingly weakened. Furthermore, spontaneous AT particle microcracking occurred as the AT content increased to 40 vol.%. The occurrence of microcracking can relieve the stress energy in the AT phase. Both effects contributed to the remarkable decrease in tensile stresses in the AT phase of the A–AT composites with increasing AT content.

No significant change was detected in the stress value of the Al_2_O_3_ matrix as the addition of second-phase AT increased. This was inconsistent with the trend in other alumina-based ceramic composites reinforced by the second phase with higher thermal expansion, showing an increase in the absolute value of compression in the Al_2_O_3_ matrix [33,48]. This could be attributed to the special microcracking characteristics of AT particulates in A–AT ceramics. As AT vol.% increased, more microcracks were generated in AT and propagated along the boundaries of Al_2_O_3_ and AT. Microcracking benefited the absorption of stress energies during the expansion and shrinkage of materials during fabrication. Consequently, the stress state of the Al_2_O_3_ matrix in the A–AT composites was not significantly affected by the increase in AT content. This effect was also demonstrated by the thermal expansion curve of A–AT composites reported in a previous study [49], showing significant thermal expansion hysteresis in A-40AT compared with the A-10AT composites. 

After sintering, temperature changes during cooling can lead to a higher CTE misfit strain value, thus increasing stresses. However, our measurement showed lower tension in the AT phase in the A–AT composites with higher sintering temperatures. Microcracking was presumed to be responsible for this inconsistency. Based on Figure 3 and Table 2, at higher sintering temperatures, the grain sizes of both AT and Al_2_O_3_ increase, resulting in further microcracking as a higher population of AT grains surpasses its critical size. This contributes to the release of thermal stress energy from samples with higher sintering temperatures; consequently, the tension in the AT phase decreases.

Moreover, according to the derived mean phase stresses of the Al_2_O_3_ matrix and AT particles derived by neutron diffraction, the macro-residual stresses, $\sigma_{bulk}$, of the A–AT bulk samples were calculated using the following equation:

(11) $$\sigma_{bulk}=f_{A}\sigma_{A}+f_{AT}\sigma_{AT}$$

where $f_{A}$ and $f_{AT}$ are the volume fractions of Al_2_O_3_ and AT, respectively; and $\sigma_{A}$ and $\sigma_{AT}$ are the mean phase stresses of Al_2_O_3_ and AT, respectively.

The through-thickness values, $\sigma_{bulk}$, of the A–AT bulk samples are plotted in Figure 7. Macro-residual stresses, $\sigma_{bulk}$, were initially assumed negligible in all the A–AT bulk samples because no pressure was applied during the sintering process. However, according to the results, the $\sigma_{bulk}$ values were not zero but compressive in the A–AT samples. This could be explained by considering the unknown stress states near the surface areas. As mentioned, strain scanning using neutron diffraction measurements was implemented 2 mm away from sample surfaces. The stress state near the surface was unknown because it could not be determined by neutron diffraction. Based on the hydrostatic assumption, tensile stress was anticipated around the surface area of the A–AT bulk.

#### 3.4.2. Peak-Specific Residual Stresses

Peak-specific residual stresses were assessed by measuring the shifts in individual diffraction peaks in the TOF diffraction spectrum. Considering the peak intensity, as well as the non-overlapping and clear shape in the diffraction spectra, the peaks of Al_2_O_3_ (i.e., (030), (116), (024), (113)) and AT (i.e., (243), (200), (043), and (113)) were selected for analysis, as shown in Figure 6. The d-spacing of each peak was obtained by Rietveld refinement. Peak-specific residual stresses for the selected peaks were calculated using Equations (9) and (10).

**Figure 8:**
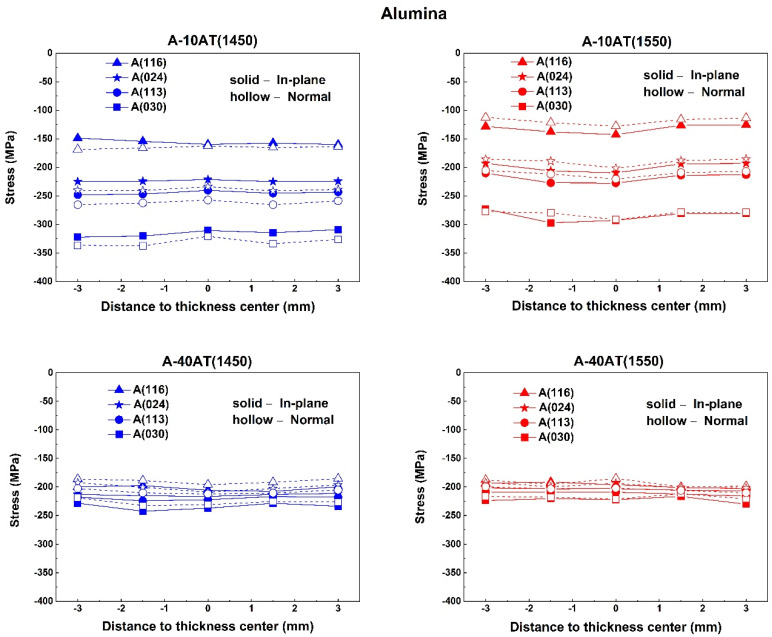
Through-thickness residual stress profiles corresponding to alumina reflections (i.e., (116), (024), (113), and (030)) in Al_2_O_3_–Al_2_TiO_5_ composites (1450 °C in blue line and 1550 °C in red line) measured along inplane (solid line and solid symbol) and normal (dash line and hollow symbol) directions.

**Figure 9:**
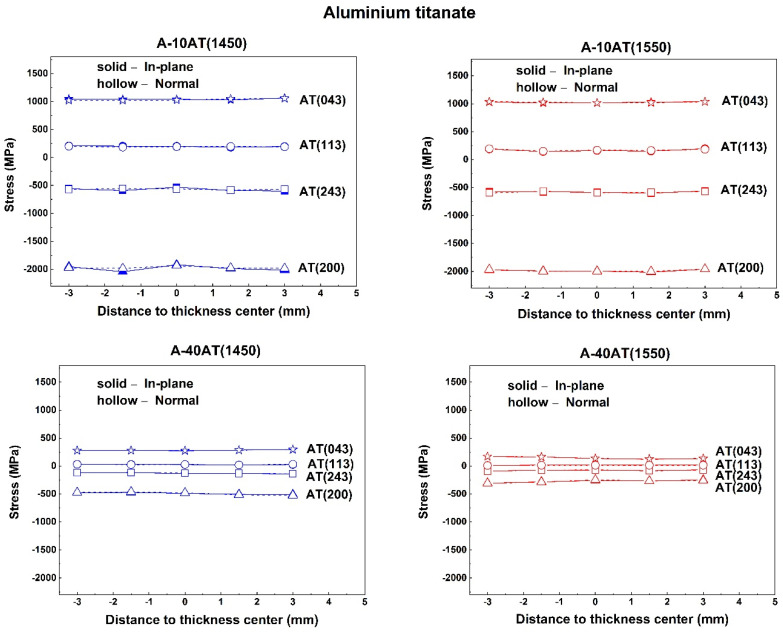
Through-thickness residual stress profiles corresponding to AT reflections (i.e., (043), (113), (243), and (200)) in Al_2_O_3_–Al_2_TiO_5_ composites (1450 °C in blue line and 1550 °C in red line) measured along in-plane (solid line and solid symbol) and normal (dash line and hollow symbol) directions.

The through-thickness peak-specific residual stress behaviors of selected reflections of Al_2_O_3_ and AT in all samples, measured in both the in-plane and normal directions, are plotted in Figure 8 and Figure 9, respectively.

As shown in Figure 8 and Figure 9, the through-thickness residual stress profiles of all the selected reflections are virtually flat in both the in-plane and normal directions, confirming the occurrence of mean phase stress behaviors as previously discussed. Variations among peak-specific residual stresses are not remarkable between the in-plane and normal directions. The average peak-specific residual stress values of the selected peaks for both the Al_2_O_3_ and AT phases are summarized in Table 4 and Table 5, respectively; these values were obtained regardless of the measurement directions. To clearly contrast the stresses of the Al_2_O_3_ and AT phases, their average mean phase stresses are also listed.

For the same phase in the same sample, the obtained peak-specific residual stress values varied among different hkl reflections. Compression was developed in all the selected hkl reflections of the Al_2_O_3_ phase in the studied A–AT composites. However, the AT phase underwent tension in the reflections of (043) and (113); compression occurred in (243) and (200). The values of the peak-specific residual stresses in each phase differed from their mean phase stresses. As the AT content increased from 10 to 40 vol.%, the variation ranges of residual stresses in different reflections became smaller for both the Al_2_O_3_ and AT phases. However, the orders of peak-specific residual stress values in different hkl reflections were unchanged in each phase.

The effects of different sintering temperatures on peak-specific residual stresses were investigated. With a higher sintering temperature, the A-10AT composites exhibited reduced absolute values of compression in all selected hkl reflections of Al_2_O_3_; however, no distinct difference in peak-specific residual stresses of the AT phase was observed. Conversely, in the A-40AT composites, the peak-specific residual stresses in the Al_2_O_3_ phase between A-40AT(1450) and A-40AT(1550) were similar, whereas the AT phase exhibited evident differences in peak-specific residual stresses between these two samples. With a higher sintering temperature in the A-40AT composites, the variation range of residual stresses in different hkl reflections were reduced in the AT phases.

The results of peak-specific residual stresses, calculated from the d-spacing of each reflection, can be explained by the lattice parameter values. For hexagonal α-Al_2_O_3_, the relationship between the d-spacing (*d_hkl_*) for a given hkl plane and the lattice parameters, a (a=b) and c, is written as follows [50]:

(12) $$d_{hkl}=\frac{1}{\sqrt{\frac{4}{3a^{2}}\left(h^{2}+k^{2}+hk\right)+\frac{l^{2}}{c^{2}}}}$$

According to the obtained lattice parameters listed in Table 3, the a and c axes of α-Al_2_O_3_ shrunk in all the studied A–AT composites when compared with those of the Al_2_O_3_ reference powder. Thus, according to Equation (12), for a given lattice plane hkl of Al_2_O_3_, the d-spacing decreased, and compression developed in all the selected reflections in the Al_2_O_3_ phase in the A–AT composites.

As the AT content increased from 10 to 40 vol.%, the a axis of Al_2_O_3_ slightly expanded, but the c axis shrunk. This led to peak-specific residual stresses in some hkl reflections and a certain reduction in the Al_2_O_3_ phase. For example, for hk0 planes in the Al_2_O_3_ phase in the A-40AT composites, with a slightly increased a value, $d_{hk0}$ increased, and the absolute values of the compressive peak-specific residual stresses decreased compared with those in the A-10AT composites. This is consistent with the measured residual stresses of A(030) in the A–AT composites, as listed in Table 4.

Compared with the A-10AT(1450) composites, the A-10AT(1550) composites showed slight increases in the a and c axes of α-Al_2_O_3_. Thus, with a higher sintering temperature value, the d-spacing of each Al_2_O_3_ reflection slightly increased, and the absolute values of compressive peak-specific residual stresses decreased. In the A-40AT composites, the difference in the lattice parameters of Al_2_O_3_ was not distinct at different sintering temperatures; thus, no remarkable difference was observed in the peak-specific residual stresses of Al_2_O_3_ in the two samples.

For orthorhombic AT, with the lattice parameters a, b, and c, the interplanar spacing ($d_{hkl}$) is given by the following:

(13) $$d_{hkl}=\frac{1}{\sqrt{\frac{h^{2}}{a^{2}}+\frac{k^{2}}{b^{2}}+\frac{l^{2}}{c^{2}}}}$$

As summarized in Table 3, the a axis of the AT phase shrunk, and the b and c axes expanded in all the studied A–AT composites compared with those of the AT reference powder. Thus, for a specific hkl plane of AT, the d-spacing may decrease or increase according to Equation (13). This leads to peak-specific residual stresses, either compression or tension in different selected reflections in AT. For example, for the 0kl reflections, $d_{0kl}$ increased, and tension developed. For the *h*00 reflections of AT, $d_{h00}$ decreased, and compression developed.

As the AT content increased from 10 to 40 vol.%, the a axis of the AT phase expanded, and the b and c axes shrunk. Thus, for the 0kl reflections in AT, the d-spacing decreased, and tension decreased. For the *h*00 reflections in AT, $d_{h00}$ increased, and the absolute values of compression decreased. These are all in accordance with the measured peak-specific residual stress behaviors of AT(043) and (200), as summarized in Table 5.

With regard to the effect of different sintering temperatures, owing to the similar lattice parameters of AT in the A-10AT(1450) and A-10AT(1550) samples, the peak-specific residual stresses for each selected AT reflection were similar in both samples. In the A-40AT composites, as the sintering temperature increased from 1450 to 1550 °C, the a axis of the AT phase expanded, and the b and c axes shrunk. Consequently, for AT(430) in A-40AT(1550), $d_{043}$ decreased, and the tension decreased with respect to that in A-40AT(1450). For AT(200) in A-40AT(1550), $d_{200}$ increased; consequently, the absolute compression value decreased with respect to that in A-40AT(1450).

Owing to the strong anisotropy of thermal expansion in the AT phase, with higher AT content and sintering temperature, more microcracks are generated in the A–AT composites. This leads to the reduced connectivity of the material in the composites; thus, the restraints in the material are weakened. Hence, the anisotropy of peak-specific residual stresses in both Al_2_O_3_ and AT phases was weaker in A-40AT than in the A-10AT composites, and the anisotropy of the peak-specific residual stresses of the AT phase was weaker in A-40AT(1550) than in A-40AT(1450).

As mentioned above, it is well accepted that peak-specific residual stress behaviors are mainly determined by the crystal structure of each phase; this is closely related to the CTE anisotropy in each phase and the microstructure of materials. On the one hand, owing to the anisotropy of thermal expansions and elastic properties in each hkl direction, for the same sample and phase, the residual stress values obtained vary from different reflections. The sign and magnitude of residual stress values considerably depend on the reflection used for analysis in the diffraction method. This is an important concern in residual stress measurements and analysis using the diffraction method with single-peak reflections in complex materials. On the other hand, with looser microstructures, such as microcracking, the anisotropy of peak-specific residual stresses in each phase was distinctly weakened. Deriving reliable properties is beneficial owing to lower internal stresses. However, a looser structure may limit the strength of materials. Thus, to balance the weakening of the anisotropy of residual stresses and improve mechanical properties, optimizing and controlling the microstructure of materials are crucial.

## 4. Summary and Conclusions

In summary, a series of Al_2_O_3_–Al_2_TiO_5_ bulk composites was investigated, with different AT contents (10 and 40 vol.%) and fabricated with different sintering temperature (1450 and 1550 °C) in order to develop significantly different microstructural features. The microstructure, crystal structure, and through-thickness residual stress profiles of the samples were obtained.

The main conclusions of the present study are as follows:

(1) The sintering temperatures of 1450 °C ensured the complete formation and retention of Al_2_TiO_5_ during the reaction sintering and the subsequent cooling of the A–AT composites. Some decomposition of AT occurred in the A–AT composites at the sintering temperature of 1550 °C.
(2) The increase of AT content (from 10 to 40 vol.%) and sintering temperature (from 1450 to 1550 °C) resulted in microstructure evolution in the A–AT composites, from a dense fine-grained microstructure to coarse microstructure with AT grain growth and microcracking. Therefore, the flexure strength was correspondingly decreased.
(3) The lattice parameters of both Al_2_O_3_ and AT varied with the AT content and sintering temperature in the studied A–AT composites, mainly because of thermal strains.
(4) Owing to the CTE mismatch between the matrix and particles, tensile residual stresses developed in the AT particulates and compressive stresses in the Al_2_O_3_ matrix, with almost flat through-thickness residual stress profiles. The increase of AT content and sintering temperature led to the change of the mean phase stress of both AT and Al_2_O_3_ in the composites. Microcracking is an important factor of residual stress, contributing to the release of thermal stress energy in samples.
(5) Owing to the thermal expansion anisotropy in each phase, significant stresses built up in different hkl reflections for both the Al_2_O_3_ and AT phases. The peak-specific residual stress profiles of both the Al_2_O_3_ matrix and AT particulates were virtually flat throughout the sample thickness, with stress values varying along different hkl reflections. The sign and magnitude of residual stress values considerably depend on the reflection used for analysis in the diffraction method. This is an important concern in residual stress measurements and analysis using the diffraction method with single-peak reflections in complex materials.

## Figures and Tables

**Table 1:** Initial crystal structure parameters of Al_2_O_3_ and Al_2_TiO_5_ phase used in refinement.

Phase	Space Group	Lattice Parameter/Å	Ion Position and Coordinate			
			Ion	x	y	z
α-Al_2_O_3_ [31] (hexagonal)	$R\bar{3}$ *c*	a=b = 4.7602 c = 12.9933 α =β = 90° γ = 120°				
			Al^3+^	0	0	0.35216
			O^2−^	0.30624	0	0.25
			
Al_2_TiO_5_ [13] (orthorhombic)	*Cmcm*	c = 3.593 a = 9.433 b = 9.641 α =β =γ = 90°	Ti_1_^4+^	0	0.1854	0.25
			Al_1_^3+^	0	0.1854	0.25
			Ti_2_^4+^	0	0.13478	0.5615
			Al_2_^3+^	0	0.13478	0.5615
			O_1_^2−^	0	0.7577	0.25
			O_2_^2−^	0	0.0485	0.1167
			O_3_^2−^	0	0.3125	0.0721

**Table 2:** Values of measured density (*ρ*), relative density (*ρ*_relative_), and average grain size (*G*) of Al_2_O_3_ and Al_2_TiO_5_, and flexural strength (*σ*_f_).

Sample	Al_2_TiO_5_ (vol.%)	Sintering Temperature (°C)	*ρ* (g/cm^3^)	*ρ*_relative_ (T.D.%)	*G* (μm)		*σ*_f_ (MPa)
					*G*_A_	*G*_AT_	
A-10AT(1450)	10	1450	3.86 ± 0.03	97.4 ± 0.1	3.1 ± 0.4	1.3 ± 0.4	221 ± 8
A-10AT(1550)	10	1550	3.84 ± 0.03	97.0 ± 0.1	3.4 ± 0.4	1.6 ± 0.4	194 ± 9
A-40AT(1450)	40	1450	3.57 ± 0.06	92.1 ± 0.2	1.7 ± 0.4	3.0 ± 0.7	83 ± 3
A-40AT(1550)	40	1550	3.53 ± 0.08	91.2 ± 0.2	2.3 ± 0.7	4.1 ± 0.9	61 ± 2

**Table 3:** Average lattice parameters (A°) of Al_2_O_3_ and Al_2_TiO_5_ phases obtained by Rietveld refinement of initial powders and A–AT composite samples.

Sample	α-Al_2_O_3_ Phase				Orthorhombic Al_2_TiO_5_ Phase					
	In-Plane		Normal		In-Plane			Normal		
	a	c	a	c	a	b	c	a	b	c
Al_2_O_3_ powder	4.76026	12.99488	4.76048	12.99456	-	-	-	-	-	-
Al_2_TiO_5_ powder	-	-	-	-	3.59348	9.43079	9.63653	3.59375	9.43032	9.63763
A-10AT (1450)	4.7585 (1)	12.9940 (3)	4.7582 (1)	12.9937 (3)	3.5643 (9)	9.4529 (10)	9.7118 (8)	3.5649 (4)	9.4527 (12)	9.7120 (10)
A-10AT (1550)	4.7585 (1)	12.9947 (2)	4.7588 (1)	12.9944 (1)	3.5641 (3)	9.4524 (8)	9.7112 (12)	3.5646 (9)	9.4520 (14)	9.7118 (12)
A-40AT (1450)	4.7588 (1)	12.9920 (2)	4.7591 (1)	12.9920 (4)	3.5863 (3)	9.4385 (9)	9.6534 (6)	3.5867 (4)	9.4388 (5)	9.6540 (10)
A-40AT (1550)	4.7589 (1)	12.9920 (2)	4.7592 (1)	12.9920 (4)	3.5894 (5)	9.4347 (8)	9.6456 (8)	3.5895 (5)	9.4349 (5)	9.6464 (10)

**Table 4:** Average residual stress values of selected peaks for Al_2_O_3_ compared with corresponding mean phase stresses.

Sample	Mean Phase Stress σ_Al2O3_ (MPa)	Peak-Specific Residual Stress (MPa)			
		Al_2_O_3_ (030)	Al_2_O_3_ (116)	Al_2_O_3_ (024)	Al_2_O_3_ (113)
A-10AT (1450)	−208 ± 7	−324 ± 12	−160 ± 6	−232 ± 8	−253 ± 9
A-10AT (1550)	−176 ± 6	−283 ± 7	−125 ± 9	−195 ± 8	−215 ± 7
A-40AT (1450)	−194 ± 6	−230 ± 6	−196 ± 7	−207 ± 8	−14 ± 7
A-40AT (1550)	−183 ± 7	−219 ± 4	−195 ± 5	−201 ± 4	−208 ± 5

**Table 5:** Average residual stress values of selected peaks for AT phase compared with corresponding mean phase stresses.

Sample	Mean Phase Stress σ_AT_ (MPa)	Peak-Specific Residual Stress (MPa)			
		AT (243)	AT (200)	AT (043)	AT (113)
A-10AT (1450)	576 ± 30	−572 ± 20	−1977 ± 34	1041 ± 10	195 ± 8
A-10AT (1550)	518 ± 29	−579 ± 10	−1985 ± 18	1027 ± 9	171 ± 16
A-40AT (1450)	166 ± 11	−128 ± 8	−493 ± 21	282 ± 7	27 ± 4
A-40AT (1550)	74 ± 8	−79 ± 7	−274 ± 18	145 ± 15	14 ± 2

## Data Availability

Some or all data generated or used during the study are available from the corresponding author by request.
